# Delineation of 2q32q35 Deletion Phenotypes: Two Apparent “Proximal” and “Distal” Syndromes

**DOI:** 10.1155/2013/823451

**Published:** 2013-06-09

**Authors:** Adrian Mc Cormack, Juliet Taylor, Nerine Gregersen, Alice M. George, Donald R. Love

**Affiliations:** ^1^Diagnostic Genetics, LabPlus, Auckland City Hospital, P.O. Box 110031, Auckland 1148, New Zealand; ^2^Genetic Health Service New Zealand-Northern Hub, Auckland City Hospital, Private Bag 92024, Auckland 1142, New Zealand; ^3^School of Biological Sciences, University of Auckland, Private Bag 92019, Auckland 1142, New Zealand

## Abstract

We report on three patients with interstitial deletions of the long arm of chromosome 2 involving bands 2q32.1–q35. They presented with wide-ranging phenotypic variation including facial dysmorphisms, cleft palate, learning difficulties, behavioural issues and severe heart defects. Microarray analysis confirmed an 8.6 Mb deletion in patients 1 and 2 and a 24.7 Mb deletion in patient 3. We discuss the genes involved in the deleted regions including *MYO1B*, *GLS*, *FRZB*, *SATB2*, and *CPS1* and compare the phenotype with those reported in the literature. Taken together, these data suggest that there is a spectrum of disease severity such that patients with deletions encompassing the region of 2q32.1q32.2, which includes the *FRZB* gene, show an apparently milder phenotype compared to those that lie further distal in 2q32.3q35 that encompasses the *SATB2* gene.

## 1. Introduction

Interstitial deletions of the long arm of chromosome 2 involving the 2q31q33 region are responsible for a number of clinical features, including facial dysmorphism, developmental delay, failure to thrive, mental retardation, and behavioural disturbances [1, 2]. There have been approximately 40 patients described in the literature, but a significant number of these cases are not highly resolved, so genotype-phenotype correlations have been difficult to make. More recently, molecular karyotyping has allowed high-resolution characterisation of these deletions. Van Buggenhout et al. [3] have described four independent patients and reported a recognisable pattern of clinical anomalies. Rifai et al. [4] and Cocchella et al. [1] reported two further cases, with the latter refining a critical region for the syndrome and identifying candidate genes to explain the phenotype [5].

The study described here reports on three further cases involving the interstitial region 2q32q35. Patient 1 and her mother, patient 2, carry an 8.6 Mb heterozygous deletion of region 2q32.1q32.3. Patient 3 carries a 24.7 Mb heterozygous deletion involving the region 2q32.2q35.

## 2. Clinical Report

### 2.1. Patient 1

**Figure 1:**
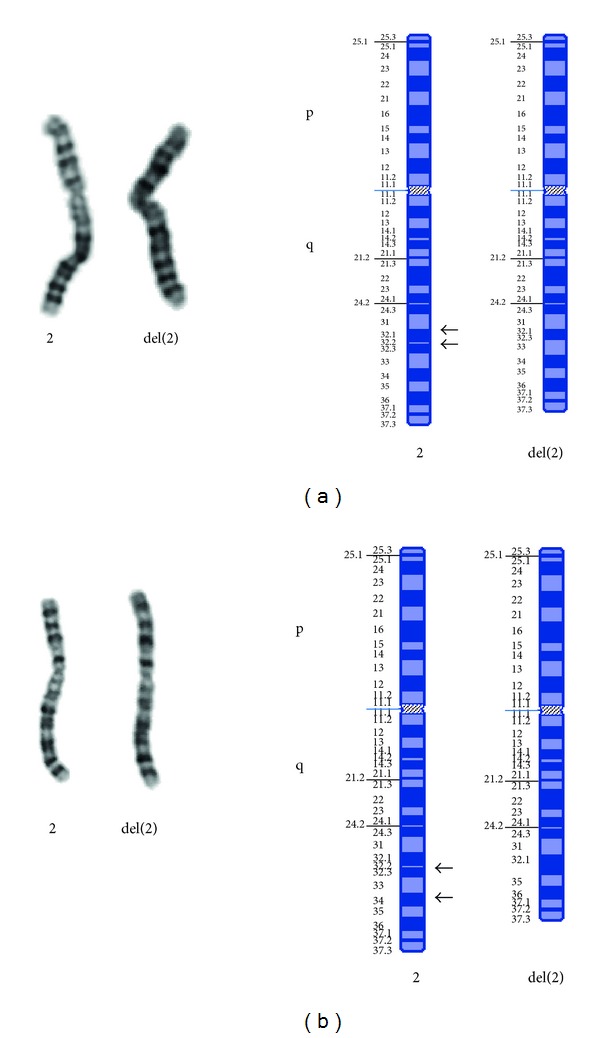
G-banded karyotype and corresponding ideogram of chromosomes 2. Panels (a) and (b) show the deleted chromosomes 2 for patients 1 and 3, respectively. The left hand of each panel shows the G-banded chromosomes, and the right side shows the corresponding ideograms. The horizontal black arrows show the location of the deletion breakpoints.

The proband (patient 1) was the first child to a nonconsanguineous couple. The antenatal ultrasound scan was normal at 21-week gestation. At 29 + 4 weeks, a clot was seen in the ductus arteriosus, resulting in poor right ventricular function and hydrops fetalis; no structural malformations were observed. An amniocentesis was performed and showed a normal male karyotype. The baby was delivered by emergency caesarean section at 29 + 4 weeks. Despite attempts at resuscitation, the baby died an hour after delivery. Postmortem examination confirmed that there were no primary malformations and also confirmed almost the complete occlusion of the ductus arteriosus by a calcified thrombus, suggesting that it had been present for at least a few weeks. High-resolution G banded analysis of a blood sample taken at the time of delivery showed a karyotype of 46,XY,del(2)(q32.2q33.1) see Figure 1(a). Parental blood samples showed that the abnormality had been maternally derived (data not shown).

### 2.2. Patient 2

The mother (patient 2) of the proband (patient 1) was born with left-sided hemifacial microsomia and underwent a number of corrective surgical procedures resulting in very limited jaw opening. She had learning difficulties throughout school and a confirmed diagnosis of obsessive compulsive disorder. She reports easy bruising but has normal wound healing. On examination, she had significant left-sided hemifacial microsomia, soft and hyperextensible skin, and evidence of hyperextensible joints. Her teeth and palate were normal. There was no significant family history of learning or behavioural difficulties, clefting, or symptoms suggestive of connective tissue disorders.

### 2.3. Patient 3

The proband (patient 3) was the first child born to nonconsanguineous New Zealand Maori parents. There was a family history of congenital heart disease (coarctation of the aorta) in a maternal uncle but no other history of congenital anomalies or intellectual disability. The pregnancy was uncomplicated apart from slowing of growth in the third trimester, which was monitored clinically. The child was born at term by spontaneous vaginal delivery with a birth weight of 2700 g (3rd centile), head circumference 33 cm (5th centile) and crown-heel length 47 cm (5th centile) and Apgar scores of eight at 1 and 5 minutes. Soon after birth she developed cyanosis in addition to respiratory distress and was admitted to the neonatal intensive care unit for respiratory support. Examination showed dysmorphic features of small palpebral fissures, telecanthus, small mouth, cleft palate, micrognathia, preauricular tags, and minor external ear anomalies. The left hip was unstable.

Echocardiograms showed a membranous ventricular septal defect, a small secundum atrial septal defect, small PDA, and severe right ventricular volume overload and persisting pulmonary hypertension. Appearances on the cranial ultrasound were suggestive of deficiency of the posterior portion of the corpus callosum, but on MRI the corpus callosum appeared to be normal, and there were no other structural abnormalities. A renal tract ultrasound was normal. Bilateral cataracts were noted on ophthalmological examination. Hip ultrasound showed a normal right hip and developmental dysplasia of the left hip. A laryngoscopy and bronchoscopy were done to investigate stridor and found a short epiglottis. 

The patient required ongoing intensive care management with continuous positive airway pressure and supplemental oxygen due to respiratory failure. She was not able to tolerate oral feeds and required nasojejunal feeds because of the risk of aspiration. At the age of 10 weeks she underwent cardiac surgery to repair her ventricular septal defect.

High-resolution G-banded analysis of a peripheral blood sample from the five-week old baby showed a karyotype of 46,XX,del(2)(q31q33) see Figure 1(b). Paternal blood was subsequently analysed and was found to be normal (data not shown).

### 2.4. Molecular Karyotype Analysis

An EDTA blood sample of all patients was requested for molecular karyotyping in order to determine the extent of the loss of chromosome 2 material and to provide more informed genetic counselling. Genome-wide copy number analysis was undertaken for patients 1 and 2 using an Affymetrix Cytogenetics Whole-Genome 2.7 M array, while the analysis of patient 3 used an Affymetrix CytoScan 750 K Array, according to the manufacturer's instructions. Regions of copy number change were determined using the Affymetrix Chromosome Analysis Suite software (ChAS) v.1.0.1 (patients 1 and 2) and v.1.2.2 (patient 3) and interpreted with the aid of the UCSC genome browser (http://genome.ucsc.edu/; Human Feb. 2009 GRCh37/hg19 assembly).

**Figure 2:**
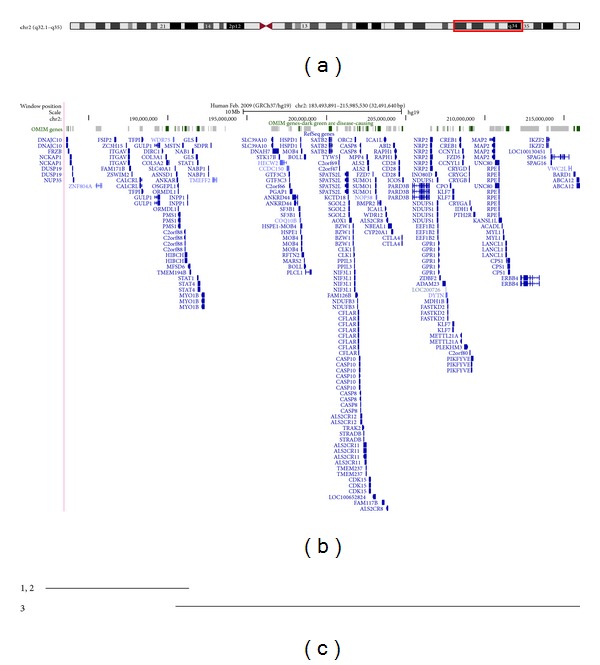
Schematic of the chromosome 2 region encompassing the deletions detected in the patients reported here. Panel (a) shows an ideogram of chromosome 2. Panel (b) shows the OMIM genes and RefSeq genes that lie in the region of the deletions reported here. Panel (c) shows the location and extent of the deletions for patients 1, 2, and 3. The images in panels (a) and (b) are taken from the UCSC genome browser (http://genome.ucsc.edu/).

The array analysis confirmed the initial cytogenetic findings and refined the breakpoints in both cases. Patients 1 and 2 carried an 8.6 Mb heterozygous deletion involving the interstitial chromosome region 2q32.1q32.3 (hg19 coordinates chr2: 183,493,891-192,126,191). Patient 3 carried a 24.7 Mb heterozygous deletion involving the interstitial chromosome region 2q32.2q35 (hg19 coordinates chr2: 191,306,412-215,985,530) see Figure 2.

## 3. Discussion

Interstitial deletions involving the 2q31q33 region have been described previously. This study examines three additional cases: a familial 8.6 Mb deletion 2q32.1q32.3 in a patient with left-sided hemifacial microsomia, learning disability and psychiatric issues, and a *de novo* 24.7 Mb deletion of 2q32.2q35 in a patient with heart defects, cleft palate, and significant dysmorphic features. 

### 3.1. Patients 1 and 2

Molecular characterisation showed a proximal breakpoint on chromosome 2 at 183 Mb and a distal breakpoint at 192 Mb. Cases have been reported previously with deletions that entirely encompass this deleted region, while other cases show varying degrees of overlap with the deleted region [1, 4–8] see Table 1. The common clinical features include learning difficulties, facial dysmorphism, and behavioural issues.

The deleted region contains 40 genes (Figure 2), but only a few are thought to be clinically significant. There are a number of possible candidate genes for the behavioural phenotype including the *GLS* gene (OMIM 138280), encoding the major enzyme involved in converting glutamine to glutamate. The significance of this enzyme derives from its possible implication in behavioural disturbances in which glutamate acts as a neurotransmitter [9]. In addition, the *MYO1B* gene (OMIM 606537) encodes a protein that participates in processes critical to neuronal development and function such as cell migration, neurite outgrowth, and vesicular transport [10]. 

The genes implicated in the defined craniofacial abnormalities of patient 2 are less clear. Cleft or high palate and facial asymmetry or craniofacial malformation have been ascribed to haploinsufficiency of the *SATB2* (OMIM 608148) and *SUMO1* genes (OMIM 601912) [11, 12], but both of these genes map immediately proximal of the deleted region. A more likely candidate is the *FRZB* gene (OMIM 605083), encoding for frizzled-related protein (FRPS), which is an antagonist of Wnt8-signalling pathway and is involved in limb and craniofacial skeletogenesis [13].

Interestingly, the deletion in patients 1 and 2 encompasses the *MSTN* gene (OMIM 601788), which is a negative regulator of muscle growth in mammals, and loss-of-function mutations are associated with increased skeletal muscle [14]. Schuelke et al. [15] reported a *MSTN* gene mutation in a mother-child couple with hypertrophic muscles. Prontera et al. [8] have argued that the “muscular phenotype” could be more pronounced in cases displaying 2q31.2q32.3 deletion compared to those with more distal deletions including del(2)(q32.2q33). These workers have considered that a muscular build pattern found in their patient may be a useful clinical handle to identify individuals with this syndrome. However, similar to the case reported by Mencarelli et al. [6], and unlike the case described by Prontera et al. [8], patient 2 does not show defined muscular presentation. 

Finally, the *COL3A1* (OMIM 12018) and *COL5A2* (OMIM 120190) genes lie in the deleted region, which encode for collagen subunits. Loss-of-function mutations of these genes are responsible for Ehlers-Danlos syndrome (EDS) types I, III, and IV [16–18]. A previous case report showed four adult patients in which haploinsufficiency of the *COL3A1* gene was associated with susceptibility of vascular aneurysms and arterial rupture [19]. While patient 2 does show some mild features of EDS, she does not show any evidence of vascular disruption and is not currently being investigated for EDS.

### 3.2. Patient 3

This case represents the second largest deletion of the 2q32 region reported thus far in the literature with proximal and distal breakpoints that map to the interval 191 Mb to 215 Mb, which carries a large number of genes. Some previously reported cases carry deletions that entirely encompass the deleted region of patient 3 [3, 7, 20], while others overlap with the deleted region [4, 5, 21, 22]. Common phenotypic features of cases with deletions that map entirely within the interval found in patient 3 are facial dysmorphism, cleft palate, heart defects, micrognathia, ear abnormalities, and feeding difficulties (Table 1). The deleted segment contains a number of genes that have been linked with these clinical presentations. The most important of these is the *SATB2* gene (OMIM 608148) which encodes for a DNA-binding protein. FitzPatrick et al. [23] demonstrated that translocations of 2q33.1 in two individuals with cleft palate were likely to disrupt the expression of the *SATB2* gene. Haploinsufficiency of the *SATB2* gene is the most likely cause of cleft palate in these translocation patients. Britanova et al. [12] also demonstrated that *Satb*2^+/−^ and *Satb*2^−/−^ mice have defects in jaw and palate morphogenesis. It has been suggested that haploinsufficiency of the *SATB2* gene accounts for developmental delay, cleft/high arched palate, and possibly even the facial dysmorphism and psychiatric problems [24].

Of the other genes within the deleted region of patient 3, the *CPS1* gene (OMIM 265380) has been linked to familial persistent pulmonary hypotension of the newborn [25]. Loscalzo et al. [22] reported a case of del(2)(q32q34) with multiple cardiac abnormalities and *CPS1* deficiency and suggested that a mutation of the remaining *CPS1* locus may account for the phenotype of their patient. While patient 3 has persistent hypotension, it is most likely caused by physical misalignment of heart vessels.

The *CRYG* gene cluster (OMIM 123660, 123670, 123680, and 123690) encodes for crystalline gamma proteins which account for one third of lens proteins. Mutations of these genes are involved in cataract formation [26]. Li et al. [27] identified a heterozygous mutation in the *crygb* gene in mice that specifically alter the subcellular distribution of gamma crystallin and results in a dense nuclear cataract. These authors have suggested that mutant crystallins can cause cataracts by selectively perturbing protein-protein interactions. Owing to the age of patient 3, a precise behavioural phenotype has not been determined although it is likely that patient will develop one based on previously reported cases. 

## 4. Conclusions

Taken together, these cases cover a region of chromosome 2 from 183,493,891-215,985,530 bp with only a small region of overlap of approximately 0.8 Mb. Table 1 shows a selection of previously published cases, and from these and other reported cases we can make a number of conclusions.

The deletion in patients 1 and 2 overlaps with others that have been reported in the literature with a common region of overlap (chr2: 181–183 Mb), which encompasses the *FRZB* gene. These cases exhibit a mild clinical phenotype despite the varying lengths of deletions. Patients with heterozygous deletions that lie in this proximal region of 2q32 (including the *FRZB* gene) could be ascribed as having a general phenotype of LD/MR, some form of craniofacial dysmorphisms and a form of behavioural disorder. Active and proper speech is rarely normal, cardiac abnormalities appear to be rare, and abnormal dentition is infrequent.

The deletion in patient 3, along with previously reported cases [3, 4, 7, 20, 21], would appear to form a more-common second set of deletions distal of the 2q32 region (including the *SATB2 *gene). These patients could be ascribed as having a more severe phenotype of LD/MR, some form of craniofacial dysmorphisms, and a form of behavioural disorder. Within these cases, active and proper speech is also rarely normal, abnormal dentition is very common, and there appears to be an increased risk of heart abnormalities.

Molecular karyotype analysis offers a much higher level of resolution than traditional cytogenetic analysis. It provides more accurate breakpoint data as well as defining the genes involved in the deleted regions. This leads to a more accurate prediction of the phenotype as well as better genetic counselling for patient. The correlation of genotype phenotype for deletions in the 2q32q35 region suggests two emerging syndromes. Critically, confirmation and resolution of these syndromes are hampered by the small number of reported cases, the age differences of patients, and the lack of detailed medical reports.

## Figures and Tables

**Table 1:** Comparison of selected patients reported with 2q32 microdeletion syndrome.

	Rifai et al. [4]	Cocchella et al. [1]	Ferreira et al. [5] Patient 1	Mencarelli et al. [6]	Patient 2	Balasubramanian et al. [7] patient 2	Patient 3	Van Buggenhout et al. [3] Patient 3	Balasubramanian et al. [7] Patient 6	Van Buggenhout et al. [3] Patient 4
Breakpoint	176,637,788–202,728,505	181,278,257–185,623,055	178,121,127–194,943,948	Approx 180,000,000–193,000,000	183,493,891–192,126,191	190,915,507–201,302,003	191,014,657–215,693,775	Approx 193,000,000–208,000,000	195,531,681–199,625,499	Approx 196,000,000–207,000,000

Age	16 Y	25 Y	8 Y 9 M	13 Y	36 Y	5 Y	6 m	3 Y 8 M	4 Y	11 Y 11 M

Mental retardation/ developmental delay	Severe intellectual disability	Severe MR	Mild MR	Severe MR	LD	Mild-moderate MR	NS	Severe	NS	Severe

Craniofacial	Asymmetric, flat malar bones, high forehead, bitemporal constriction	Long	Large narrow forehead, midface hypoplasia	Long, high forehead	Limited jaw opening, hemifacial microsomia	High forehead	Small mouth	Asymmetry	Rectangular, prominent forehead	Small

Eyes	NS	Hypertelorism, downslanting	Dacryocystitis	Deep set	NS	Downward slanting, palpebral fissures	Bilateral cataracts, telecanthus, palpebral fissures	Dacryocystitis, coloboma	Significant for hyperopia of the right eye, esotropia of the right eye	Downward slanting palpebral fissures

Ears	NS	Retrorotated	NS	Dysmorphic right ear	NS	Mild unilateral HL	Minor external ear abnormalities, prelauricular tags	Frequent otis media	Recurring otis media	Dysplastic

Teeth	Oligodontia	NS	NS	Broad, overcrowded, abnormal	Normal	Crowded teeth	NS	Abnormal	Dental crowding	Abnormal adult teeth

Palate	Soft, bifid uvula	High	High and narrow	High	Normal	High	Cleft	High	Narrow and high arched	High

Micrognathia	NS	Yes	NS	Yes	NS	NS	Yes	NS	NS	NS

Skin/hair	Hair woolly, sparse. Skin thin almost lipoatroppic	Thick hair, normal skin	Thick and coarse skin, dry hair	Thick coarse hair, thick eyebrows	Soft and hyperextendable skin	Fine hair	NS	Thin, sparse hair	Eczema	Thin white hair

Cardiac	NS	NS	NS	NS	NS	NS	Membranous ventricular septal defect, small secundum atrial septal defect, small PDA, and severe right ventricular volume overload	Small VSD	Heart murmur	NS

Behaviour	NS	Aggressive	Aggressive and unpredictable humour, uncontrolled eating habits	Aggressive, hyperactivity, anxiety, self mutilation	OCD	None	NS	Mood change, hyperactive, autistic like behaviour	Autistic like features, hyperactivity	No behavioural problems

Speech	No active speech	No active speech	Active speech	No active speech	Normal/active speech	A few words	NS	Donald Duck speech	10-word vocabulary	Donald Duck speech

NS: not specified; VSD: ventricular septal defect; PDA: patent ductus arteriosis; LD: learning disabilities.
